# Different Isoforms of HPV-16 E7 Protein are Present in Cytoplasm and Nucleus

**DOI:** 10.2174/1874357900802010015

**Published:** 2008-03-26

**Authors:** H Valdovinos-Torres, M Orozco-Morales, A Pedroza-Saavedra, L Padilla-Noriega, F Esquivel-Guadarrama, L Gutierrez-Xicotencatl

**Affiliations:** 1 Research Center of Infectious Diseases, National Institute of Public Health, Cuernavaca, Morelos, Mexico; 2 Biomedical Research Institute, National Autonomous University of Mexico, Mexico City, Mexico; 3 Faculty of Medicine, Autonomous University of Morelos State, Cuernavaca, Morelos, Mexico

## Abstract

The E7 protein of high risk HPV types has been found with different molecular weights, mainly because of phosphorylation, an event that changes protein charge and mobility in SDS-PAGE. Distribution of E7 protein in the cellular compartments has also been subject of debate as some groups report the protein in nucleus and others in cytoplasm. The different subcellular distribution and molecular weights reported for the E7 protein suggest the presence of isoforms. We examined this possibility by using several antibodies that recognize different epitopes on the HPV-16 E7 protein. We showed that E7 is processed in 3 isoforms with different molecular weights and isoelectric points (IEP), and described as E7a1 (17.5 kDa, IEP 4.68), E7a (17 kDa, IEP 6.18) and E7b (16 kDa, IEP 6.96). The immunofluorescense results also showed that E7 is distributed into different compartments (ER, Golgi and nucleus), which suggest the presence of other posttranslational modifications, besides phosphorylation.

## INTRODUCTION

Cervical cancer (CC) is one of the most prevalent diseases worldwide. CC is the second major cause of death in women from underdeveloped countries [1] and the main etiological factor is the human papillomavirus (HPV) persistent infection status. Over 90% of malignant carcinomas of the genital tract have HPV DNA sequences; the majority of which contain the high risk HPV-16 and 18 types which are linked to the progression of cervical lesions and CC [2, 3].

The mechanisms through which HPVs induce cell transformation have been intensively investigated recently. The most abundant viral transcripts in tumor and tumor cell lines come from the E6 and E7 open reading frames that are known to be oncogenic. These two genes from HPV are necessary and sufficient to induce HPV-mediated transformation of murine cells [4], immortalize human fibroblasts [5] and in cooperation with *ras* to transform baby rat kidney cells and primary human keratinocytes [6, 7].

The transformation capacity of E7 has been shown to be limited to the high risk HPV types. The E7 protein is a 98 amino acid phosphoprotein with a predicted molecular weight of 11 kDa, however this protein shows abnormal electrophoretic mobility in SDS-PAGE gels that generates different molecular weights from 14 to up to 21 kDa, as reported by different groups [8-10]. It has been suggested that the different molecular weights of E7 and its mobility in SDS-PAGE gels are the result of the acidic sequence localized in the aminoterminal region of the protein, together with its high hydrophobicity and high capacity to form oligomers [11-13].

Studies in high risk HPV types showed that E7 deregulates the cell cycle mainly by binding to and promoting degradation of the tumor suppressor retinoblastoma protein (pRb) [14, 15], resulting in the dissociation of pRb from E2F transcription factors and the premature cell progression into the S-phase of the cell cycle. In a similar way, E7 affects the pRb-related pocket proteins p107 and p130 [16, 17]. It has also been demonstrated that a phosphorylated E7 isoform is able to associate with the histone H1 kinase in the late G2/M phase of the cell cycle [16]. Other studies have demonstrated that E7 is capable of inducing the enzymatic activity of the α-glucosidase in the cytoplasm which degrades intracellular glucose and provides energy for cell growth and division [18].

The different activities of the E7 protein, the heterogeneous distribution in different cellular compartments (nucleus and cytoplasm) as well as the different molecular weights reported suggest the presence of different isoforms of E7. We examined this possibility by using polyclonal and monoclonal antibodies that recognize different epitopes on the HPV-16 E7 protein. We showed that E7 is processed into 3 forms (E7a1, E7a and E7b), one of which is phosphorylated. The molecular weight and the isoelectric point (IEP) as well as the cellular localization of the E7 isoforms were different. These results demonstrate that there are different isoforms of E7 in different cellular compartments and this may be important for interaction of the E7 protein with target proteins to generate the cellular transformation.

## MATERIALS AND METHODOLOGY

### Antibodies.

The monoclonal antibodies (mAbs) D11, B4, C2, A5 and G7 were prepared and characterized in our laboratory [19]. Briefly, 20 µg of purified recombinant cII-E7 protein from HPV-16 (expressed and purified from *E. coli*) were used to immunize mice 3 times every two weeks. Hybridomas were screened and selected by ELISA against a different recombinant E7 protein (MS2-E7) [20]. Further characterization was performed by immunoblot, immunoprecipitation and immunofluorescence.

The anti-E7 (C24 and C89) polyclonal antibodies were prepared in our laboratory against the cII-E7 protein purified by electro-elution. Briefly, rabbits were immunized 3 times with 50 µg of recombinant E7 protein and tested for the presence of specific E7 antibodies in the same way as mAbs.

The anti-E7 (clone ED17, C20), the anti-calnexine (clone H-70) and the anti-p21 (C-29) were from Santa Cruz Biotechnology (Santa Cruz, CA. USA). The anti-GM130 (clone 35) was from BD Biosciences (San Jose, CA. USA).

### *In Vitro *Cell Culture Conditions.

CaSki cell line (human cervical epidermoid carcinoma, naturally HPV-16 transformed), HaCaT cells (normal human keratinocytes) and Cos-7 (kidney monkey, SV40 transformed fibroblasts) were cultured in Dulbecco's Modified Eagle's medium (DMEM) supplemented with 10% fetal bovine serum (FBS) and maintained in a humidified atmosphere of 5% CO_2_/95% air, at 37°C.

### *In Vivo *Cell Labeling.

Cells were labeled with 50 μCi/ml [^35^S]-methionine and [^35^S]-cysteine (Promix, > 1000 Ci/mmol, GE Healthcare, Piscataway, NJ. USA), in methionine and cysteine free medium and 5% of dialyzed FBS in overnight labeling experiments or with 250 μCi/ml [^35^S]-methionine-cysteine for 15 min in pulse-chase experiments.

Cells labeled with [^32^P]-orthophosphate were starved in 0.2% FBS for 48 h and then placed in phosphate free medium for further 2 h, as described previously [21]. After this time, cells were incubated with 100 μCi/ml [^32^P]-orthophosphate (3000 Ci/mmol, GE Healthcare, Piscataway, NJ. USA) for 1 h (time zero). The remaining cells were incubated with 10% FBS for different times and labeled for 1 h with orthophosphate, as above, before cells were collected and lysed for immunoprecipitation as described below.

### Transient Transfection of HPV-16 E7 in Cos-7 Cells.

Cos-7 cells were transfected using the DEAE-dextran method as described previously [22]. Cells were seeded on 8 well Multitest Slide (MP Biomedical, Solon, OH. USA) and grown up to 80% confluence. Cells were transfected with 5 μg of purified pcDNA or pcDNA-E7 plasmid that contains the nucleotides 562 to 858 from HPV-16 which encodes for the E7 protein (GenBank access AF 477385) (a gift from Dr. J. Berumen, Genomic Medicine Laboratory, Hospital General, Mexico D.F.).  Cells were grown for different periods of time (0, 2, 4, 8, 16, 24, 48 and 72 h) after which the cells were fixed with p-formaldehyde and treated for immunofluorescence, as described below.

### Immunoprecipitation and SDS-PAGE.

Cells were lysed and immunoprecipitated as described elsewhere [23]. Briefly, cell monolayers were lysed in radioimmunoprecipitation assay (RIPA) buffer in the presence of protease inhibitors (Complete mini, Roche Diagnostics, Indianapolis, IN. USA). Cell lysates were cleared by centrifugation, and supernatants immunoprecipitated for 16 h at 4^o^C with 3 μg of purified IgG anti-E7 mAbs or 30 μl of anti-E7 rabbit polyclonal antibodies. The antigen-antibody complex was immunoprecipitated with Protein A-Sepharose (GE Healthcare,Piscataway, NJ. USA) for 2 h at 4^o^C. Rabbit anti-mouse coated Protein A-Sepharose was used for mAbs. The immunoprecipitates were dissolved in Laemmli loading buffer containing 100 mM dithiothreitol, followed by electrophoresis in a 15% SDS-polyacrylamide gel. Gels were treated for fluorography with the Enlighting reagent (NEN Life Sciences Products, Boston, MA. USA) and specific bands detected by exposing the gels to X-OMAT film.

### Immunoblot.

Proteins from different cell lines were extracted in RIPA buffer, as described previously [24], in the presence of protease inhibitors (Roche Diagnostics, Indianapolis, IN. USA). Total protein extracts (1 mg/ml) were incubated with the C24 polyclonal antibody and processed for immunoprecipitation as mentioned before. Immunoprecipitates were separated on 15% SDS-PAGE gels and transferred to nitrocellulose Protean membranes (0.45µm, Whatman International Ltd., Middlesex, UK), as described by Towbin and coworkers [25]. The membranes were blocked with PBS-0.5% Tween containing 10% skim milk for 30 min at room temperature. Subsequently, the membranes were incubated with a 1:200 dilution of anti-E7 polyclonal antibodies or with a 1:100 dilution of anti-E7 mAbs in PBS-0.5% Tween and 5% skim milk, overnight. Blots were washed with PBS-0.5% Tween followed by incubation for 1 h under the same conditions with the secondary goat anti-rabbit or rabbit anti-mouse IgG antibodies conjugated with horseradish peroxidase (DAKO, Carpinteria, CA. USA). The membranes were developed according with the chemiluminescence kit of Perkin Elmer (Waltham, Massachusetts, USA) manufacturer instructions. Membranes were exposed to X-OMAT film.

### Indirect Immunofluorescence.

Cells at 80% confluence were rinsed with PBS and fixed with 4% p-formaldehyde in PBS at room temperature for 20 min followed by treatment with permeabilization buffer [1% BSA in PBS containing either 3% Triton X-100 (for nuclear structures) or 0.2% saponine (for cytosolic and internal membranes)] for 20 min at 4^o^C. Subsequently, cells were incubated with anti-E7 antibodies (serum diluted 1:100 for anti-E7 polyclonal Abs and 2 ng/μl of purified IgG for anti-E7 mAbs in permeabilization buffer) for 16 h at 4^o^C. Slides were rinsed with PBS and incubated for 2 h at 4^o^C with anti-rabbit IgG conjugated with Alexa 488 (green) or anti-mouse IgG conjugated with Alexa 594 (red) (dilution 1:250 and 1:800, respectively, Molecular Probes, Carlsbad, CA. USA). Specimens were mounted in 50% glycerol in PBS and visualized under the Confocal Microscope 510 META (Carl Zeiss, Massachusetts, USA) under the Plan-Neofluor 100X /1.3 oil Ph3 lent.

### 2-dimensional Polyacrylamide Gel Electrophoresis (2D Gels).

Immunoprecipitated E7 protein was treated for isoelectrofocusing (IEF), as described previously [26] with some modifications. Briefly, E7 immunoprecipitates were dissolved in IEF sample buffer (9.5 M urea, 2% NP40, 2% ampholines 3-10, 100 mM DTT). IEF used 7% ampholines (GE Healthcare, NJ. USA) pH 3-9 in 2.5 mm diameter polyacrylimide gel tubes and fixed to the adaptor for slap gels electrophoresis apparatus (BRL Wrightsville, PA. USA). The IEF was carried for 16 h at 400 V and electrophoresis for the second dimension was run as described before. The gels were treated for fluorography as described previously.

## RESULTS

### Characterization of HPV-16 E7 Protein with Different Antibodies.

**Fig. (1):**
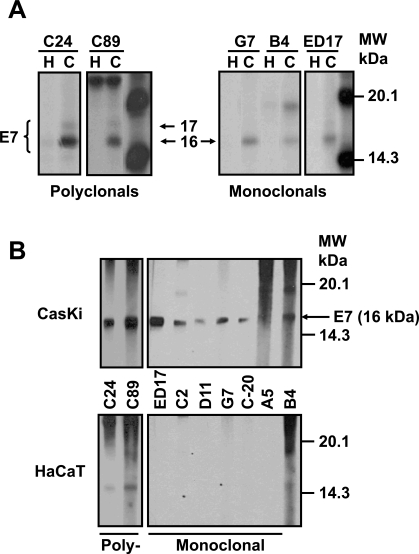
**Recognition of HPV-16 E7 protein by monoclonal and polyclonal antibodies. (A)** HaCaT (H) and CaSki (C) cells were labeled with [^35^S]-methionine-cysteine and immunoprecipitated with different anti-E7 polyclonal (C24, C89) and mAbs (G7, B4, C2, D11, C20, A5, ED17). The arrows show a 16 kDa band recognized by all antibodies, and a 17 kDa band detected only by polyclonal antibodies. **(B)** Cell extracts were first immunoprecipitated with the C24 polyclonal antibody, samples treated for Western blot and tested with polyclonal and mAbs. The arrow shows the E7 protein of 16 kDa.

It has been reported that E7 protein shows different molecular weights and that the phosphorylation process is probably one of the causes for these differences. Thus, we decided to identify the different forms reported using polyclonal and monoclonal antibodies, produced in our laboratory that can differentiate the various isoforms. To this end, CaSki cells were labeled with [^35^S]-methionine-cysteine for 16 h and the E7 protein immunoprecipitated with the different antibodies as described in Materials and Methodology. The result in Fig. (**1**) shows an E7 protein of 16 kDa that was immunoprecipitated with the polyclonal antibodies C24 and C89, as well as with the mAbs G7, B4 and the commercial ED17. However, a 17 kDa E7 protein was observed only with the polyclonal antibodies (C24 and C89) (Fig. **1A**). In contrast, none of these two bands were observed in immunoprecipitates from HaCaT cells that were used as negative controls (Fig. **1A**), or with control rabbit or mouse serum (data not shown).

We also tested the capacity of the antibodies to recognize the E7 protein by Western blot as the different molecular weights of the E7 protein could be the result of the different techniques used. Previously, we have shown that the sensitivity of the direct Western blot for E7 is low [27] and therefore, it is necessary to carry out an immunoprecipitation–Western blot to visualize the E7 protein from cell extracts. In this way, cells were immunoprecipitated first with the polyclonal C24 antibody as this antibody recognized 2 forms of E7 protein. The precipitates were separated on a SDS-PAGE gel, transferred to Protean membranes and tested with the different mAbs as described previously. The results in Fig. (**1B**) showed that 5 of the 6 mAbs tested recognized the 16 kDa band of the E7 protein. This band was also recognized by the commercial mAb ED17 acquired from Santa Cruz, which was used as a positive control. The recognition of 16 kDa band is specific, as this was not present when the mAbs were tested with the HaCaT immunoprecipitates used as negative controls (Fig. **1B**). In the case of the polyclonal antibodies (C24 and C89), they only recognized the 16 kDa band but not the 17 kDa band in the Western blot. It is possible that these antibodies only recognize a conformational epitope in the E7 protein that is lost by denaturation during the Western blot process (Fig. **1B**).

### Processing of HPV-16 E7 Protein.

**Fig. (2):**
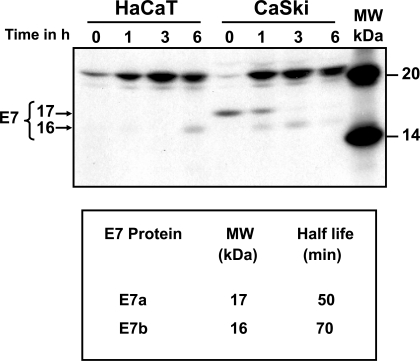
**Processing and half-life of the HPV-16 E7 protein.** HaCaT and CaSki cells were pulse labeled with [^35^S]-methionine-cysteine for 15 min and chased for different times (0, 1, 3 and 6 h). Upper panel: Cell extracts were immunoprecipitated with the anti-E7 polyclonal C89 antibody, samples separated by SDS-PAGE gel and bands visualized by auto-radiography. The arrows showed a 17 kDa (E7a) and a 16 kDa (E7b) bands. The 15 kDa band observed in HaCaT cells is a non-specific band as it is not consistently observed in experiment repetitions. Lower panel: Bands were scanned and plotted in a graph to calculate the half-life of the different forms of E7 protein.

Processing of E7 protein was determined by pulse-chase labeling of CaSki cells and immunoprecipitated with C89 polyclonal antibody (Fig. **2**). The top panel of Fig. (**2**) shows the electrophoretic analysis of immunoprecipitates of [^35^S] methionine-cysteine labeled E7 protein after 15 min pulse, followed by chase of up to 6 h. It is clear that the E7 protein was synthesized after the 15 min pulse as a 17 kDa protein (E7a) that remained stable for up to 1 h and subsequently disappeared (Fig. **2**). After 1 h of chase the E7a protein was processed to a 16 kDa band (E7b) and was stable for up to 3 h of chase (Fig. **2**). This pattern of bands was not observed in the immunoprecipitates of labeled HaCaT cells used as control (Fig. **2**). The 15 kDa band observed in HaCaT cells at 6 h (smaller than the one observed for E7 in CaSki cells) is a non-specific band as it is not consistently observed in experiment repetitions. The bands of the fluorography were scanned to calculate the half-life of the E7 proteins according to Belle and coworkers [28]. The results showed that the half-life of the E7a protein was only 50 min and 70 min for the faster moving band of E7b (Fig. **2**, lower panel). The presence of a nonspecific 21 kDa band in pulse-chase cells (HaCaT and CaSki) was due to the high levels of label used in this kind of experiments.

### Recognition of 3 Different forms of HPV-16 E7 Protein by Differences in Charge.

**Fig. (3):**
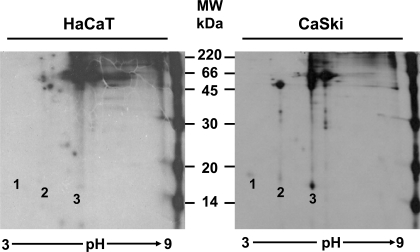
**Characterization of the HPV-16 E7 proteins by IEP.** HaCaT and CaSki cells were labeled with [^35^S]-methionine-cysteine and immunoprecipitated with the C89 polyclonal antibody. The immunoprecipitates were separated in the first dimension in a pH gradient from 3-9 for 16 h. The second dimension was run in a 15% PAGE gel and treated for fluorography. The numbers over the CaSki panel show the localization of the 3 isoforms of HPV-16 E7 proteins (E7a1, E7a and E7b) and these spots were not observed in the HaCaT control cells.

The pulse-chase experiments showed that the E7a protein was processed to a faster moving form described as E7b. At this point, it was not clear if there was an actual chemical modification of the protein, or if the differences were only due to a conformational change. For this reason, it was important to determine if there was any difference in charge in addition to the difference in molecular weight between these 2 isoforms of E7. Isoelectrofocusing of immunoprecipitates (C89 antibody) of overnight [^35^S] methionine-cysteine labeled E7 proteins was performed followed by SDS-PAGE gels. The separation of the E7 proteins in a pH gradient showed that E7a and E7b have different net charge (Fig. **3**, CaSki, spots 2 and 3 respectively). However, unexpectedly an additional form of the E7 protein in the extract of CaSki cells that was not resolved in one dimension gels was found. The third band observed (E7a1) showed a molecular weight of 17.5 kDa (Fig. **3**, CaSki, spot 1). The IEP for each form of E7 protein was calculated as 4.68 for E7a1 (spot 1), 6.18 for the E7a form (spot 2) and 6.96 for E7b (spot 3). This difference in charge among the 3 isoforms of E7 protein, suggests that the proteins are modified in some ways in two different steps.

### Phosphorylation of the HPV-16 E7 Protein.

**Fig. (4):**
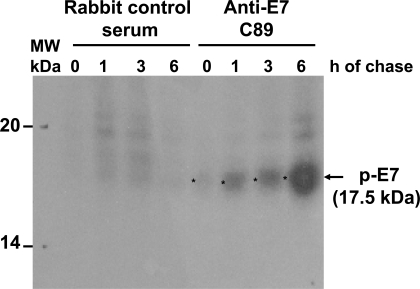
**Phosphorylation of HPV-16 E7 protein.** CaSki cells were starved for 16 h, labeled with [^32^P]-ortophosphate and harvested at different periods of time. Cell extracts were immunoprecipitated with the anti-E7 polyclonal C89 antibody or with control rabbit serum. The precipitates were separated in a 15% acrylamide gel and visualized by autoradiography. The arrow shows the phosphorylated E7 protein of 17.5 kDa.

Previously, it has been reported that posttranslation phosphorylation occurs in E7 [29]. Accordingly, we decided to identify which of the E7 forms was phosphorylated. To this end, cells were starved for 48 h and subsequently labeled with [^32^P]-orthophosphate for 1 h at different times after protein synthesis reactivation by addition of FBS as described in Materials and Methodology, and separated in 15% SDS-PAGE gel. The starvation step in this system was introduced to avoid incorporation of [^32^P]-orthophosphate in the peptide backbone and to look for novel protein phosphorylation. The result showed a phosphorylated E7 protein with an apparent molecular weight of 17.5 kDa (E7a1) that accumulates over the time, however, the 16 kDa protein band (E7b) was not observed at any time, suggesting that this E7 isoform is not phosphorylated (Fig. **4**). Several reports have shown that E7 can be phosphorylated in at least 2 different sites [21, 30, 31], and this could explain the presence of a broad band that could contain the 17.5 kDa (E7a1) and the 17 kDa (E7a) proteins that were observed in the 2D gels.

### Cellular localization of the different forms of HPV-16 E7 proteins.

**Fig. (5):**
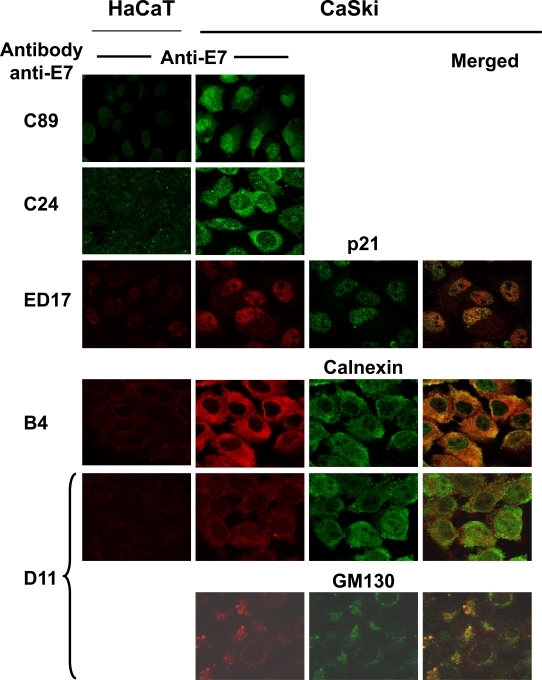
**Subcellular localization of HPV-16 E7 proteins by immunofluorescence**. Cells were fixed with p-formaldehyde and permeabilized with 0.2% saponine. Cells were tested with the different anti-E7 polyclonal (C89 and C24) and mAbs (ED17, B4, D11). Biological cell markers were used to identify ER (calnexin), Golgi (GM130) and nucleus (p21) and co-localized with the immunological detected E7 protein. Secondary fluorescent antibodies were anti-rabbit IgG conjugated with Alexa 488 (green) or anti-mouse IgG conjugated with Alexa 594 (red). The merged image is shown in the right side of the figure and positive co-localization is observed in yellow color. Images were taken at a magnification of 1000X using Confocal microscope.

Up to this point, the results showed that HPV-16 E7 from CaSki cells is processed in 3 different molecular weight forms, one of which is phosphorylated (E7a1). We also demonstrated that polyclonal and mAbs recognized different forms of the E7 protein. Taken together, this information made us wonder about the cellular localization of the 3 isoforms of E7. Thus, we used the different polyclonal and mAbs in cells fixed for immunofluorescence and co-localized the E7 protein with antibodies against cellular markers for nucleus (p21), endoplasmic reticulum (ER; calnexin) and Golgi (GM130), as previously reported [32-34]. The results in Fig. (**5**) showed that the polyclonal antibody C89 recognized the E7 protein in the nucleus as wells as in cytoplasm, while the C24 polyclonal mainly stained the cytoplasm. When mAbs were tested together with the cellular markers it was evident that the ED17 antibody recognized the E7 protein in the nuclear compartment as this antibody co-localized only with the p21 nuclear marker (Fig. **5**, ED17, merged). The E7 protein recognized by the B4 mAb co-localized only with the calnexin marker, which suggests that the E7 form recognized by this antibody is present in the ER (Fig. **5**, B4, merged). However, when the D11 mAb was tested a small part of this antibody co-localized with the calnexin marker of ER and a stronger co-localization signal was observed with the Golgi GM130 marker (Fig. **5**, D11, merged). These results demonstrated that the E7 protein is recognized in different cellular compartments by different antibodies.

### *In Vivo* Cellular Processing of HPV-16 E7 Protein in Transfected Cells.

**Fig. (6):**
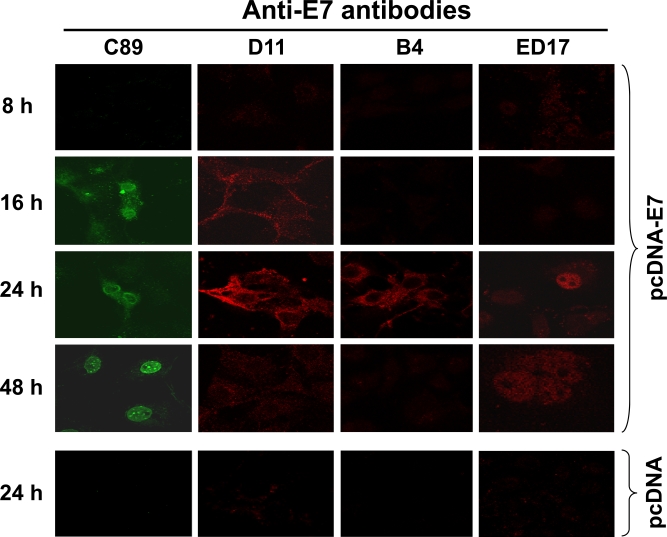
**Processing of the HPV-16 E7 protein under *in vivo* conditions.** Cos-7 cells were transiently transfected with the pcDNA-E7 plasmid and chase for different periods of time (8, 16, 24 and 48 h). Harvested cells were fixed with 4% p-formaldehyde, permeabilized as described in Material and Methodology and tested with different anti-E7 antibodies. The antibodies tested were C89 polyclonal antibody, and D11, B4 and ED17 mAbs. Secondary fluorescent antibodies were anti-rabbit IgG conjugated with Alexa 488 (green) or anti-mouse IgG conjugated with Alexa 594 (red). Cos-7 cells transfected with pcDNA plasmid alone at 24 h was used as control of the system as this was the time for the highest expression of the E7 protein observed. Images were taken at a magnification of 1000X using Confocal microscope.

The presence of different forms of E7 localized in different cellular compartments prompted us to analyze the processing of the E7 protein in the cell. To analyze the E7 processing, Cos-7 cells were transiently transfected with the pcDNA-E7 plasmid to investigate *de novo* expression and localization of E7 protein at different periods of time and tested for immunofluorescence, as described in Materials and Methodology. The results in Fig. (**6**) show that the polyclonal C89 antibody identified 3 different patterns of E7 staining; at first E7 is recognized by 16 h after transfection in the cytoplasm of the cells. By 24 h the E7 protein became concentrated in the periphery of the nucleus and by 48 h the fluorescence was only observed into the nucleus and concentrated in the nucleolus (Fig. **6**, C89). With the D11 antibody (identifies E7 in ER and Golgi) a very light cytoplasmic fluorescence signal was observed by 16 h after E7 transfection, that became very strong by 24 h (Fig. **6**, D11). However, the fluorescence signal disappeared totally by 48 h after Cos-7 cells transfection. In contrast, the B4 mAb that recognized only the E7 protein in the ER showed a strong fluorescence signal only at 24 h after E7 transfection (Fig. **6**, B4). While the ED17 mAb that recognized E7 protein only in the nucleus identified the protein only after 24 h and the fluorescence signal was still present by 48 h (Fig. **6**, ED17). In these experiments the presence of E7 expression was not detected, with any of the different antibodies tested, before the 16 h and after 48 h (data not shown). The specificity of each one of the antibodies was determined by immunofluorescence using Cos-7 transfected cells with pcDNA plasmid alone as it was done for the pcDNA-E7 plasmid (data not shown). In Fig. (**6**), only the 24 h time pcDNA control is shown for each one of the antibodies as this was the time of the highest E7 expression in the transfected cells.

## DISCUSSION

The E7 protein is a very well studied protein that had been demonstrated to interact with a variety of target proteins such as the pocket proteins (pRb, p130 and p107) [14, 15], proteins involved in cell cycle (E2F-Cyclin A complex, Cyclin E, p21^Waf1^, p27^Kip1^) [35-39], transcription factors (pCAF acetyltransferase, TBP, AP-1 family factors) [40-43] and with the senescence-regulating protein DEK [44], among others. All of these target proteins are present in the nucleus; however the reports about the distribution of the E7 in the cell have shown that this protein is present not only in nucleus but also in the cytoplasm. We believe that this differential distribution of E7 in the cells is due to the presence of different isoforms of E7 that are produced during its cellular processing. To test this hypothesis, monoclonal and polyclonal antibodies that recognize different epitopes in the E7 protein were developed in our laboratory. The results showed the presence of 3 isoforms of HPV-16 E7 protein that vary in molecular weight and IEP. These E7 proteins were described as E7a1 (17.5 kDa and IEP of 4.68), E7a (17 kDa and IEP of 6.18) and E7b (16 kDa and IEP of 6.96). The half-lives of the E7 proteins were calculated as 50 min for E7a and 70 min for E7b. These results are consistent with a previous report that showed that the half-life of E7 from CaSki cells was 55 min and 70 min in SiHa cells, although these researchers only identified one E7 band protein in each cell line [30]. The difference between Smotkin and Wettstein results and ours was the use of different anti-E7 antibodies and that these recognized the different isoforms of the E7 protein.

In the pulse-chase experiments we identified that E7 is initially synthesized as a 17 kDa protein and after approximately 1 h, it is processed to a faster moving band of 16 kDa with a short half-life. These results suggest that E7 undergoes some posttranslational modifications that generate the shift in molecular weight. A known modification for E7 is the phosphorylation of the serine residues 31 and 32 [29, 45], although serine 71 is also a potential phosphorylation site [31, 46]. Our results showed a broad phosphorylated band of a calculated molecular weight of 17.5 kDa that could be the 17 kDa band that was recognized in the immunoprecipitations. The difference in molecular weight could be due to the change in charge that makes the protein to be retarded in the SDS-PAGE gel. Another possibility is that the antibodies did not recognize efficiently the non-phosphorylated protein as has been reported for other mAbs [47], suggesting that the phosphorylated E7 protein has distinct antigenic properties (conformational changes). However, these results did not account for the lower molecular weight band of 16 kDa (E7b) that was identified in the Western blot and in the immunoprecipitations. Another as yet unidentified modification could exist and be the reason for the presence of this faster moving E7b protein.

According to the HPV-16 E7 protein sequence the calculated IEP of this protein is 4.05, however, when this characteristic was measured with recombinant E7 protein produced in bacteria the IEP obtained was 5.4 [48]. When the IEP of the HPV-18 E7 protein produced *in vitro* (using rabbit reticulocyte lysates) or immunoprecipitated from HeLa cells, there were 3 bands of the same molecular weight, but different IEP [49]. The researchers suggested that this was a typical pattern for phosphorylated proteins and this was similar to what we found. However, in our experiments not only the IEP, but also the molecular weights of the E7 proteins (E7a1, E7a and E7b), were different in the 2D gels. This again suggests that the C89 polyclonal antibody is recognizing different epitopes of the E7 isoforms that have not been fully characterized.

At least 2 distinct molecular weights have been reported for HPV-16 E7 by 2 different groups [50, 51]. However, the C89 antibody was able to recognize simultaneously 2 forms of E7 by radio-immunoprecipitation and 3 when 2D gels were used. It has been shown that E7 is highly hydrophobic and that this characteristic together with its conformational structure produce an anomalous electrophoretic behavior. Purified E7 protein is present as oligomers, and tends to be soluble and with a molecular weight close to the calculated in the presence of 8M urea [13, 52]. It is possible that we were able to visualize the 3 forms of E7 in the 2D gels, not only because the difference in charge, but because the proteins were solubilized in 8M urea.

When we examined the localization of the different E7 forms in the cellular compartments by immunofluorescence staining, it was observed that the E7 protein was first observed in the ER as this was the first signal observed with the antibodies (16 h). Subsequently, the E7 protein was visualized in the Golgi compartment (16 to 24 h) and finally translocated to the nucleus (48 h), where it probably interacts with target proteins, such as pRb. From this work, it is clear that the E7b (16 KDa, recognized by mAbs) isoform is localized in the cytoplasm and nucleus, but the localization of the 17 and 17.5 kDa E7 isoforms still needs to be determined as there are not specific antibodies available to differentiate them. According with this processing of the E7 protein, it is possible that the protein encounters some posttranslational modifications during the transit through different cellular compartments that allow the final localization of the protein. Studying the amino acid sequence of the HPV-16 E7 protein, it could be observed that it contains a consensus sequence for glycosylation of Asp 29, has 2 sulfation sites (Tyr 23 and 26) and 5 phosphorylation sites besides the already known Ser 31, 32 and 71 [46]. Up today, only phosphorylation has been reported for the E7 protein and this posttranslational modification only accounts for the difference in charge identified in the 3 different forms of E7, but other modifications will still need to be studied to clarify the differences on molecular weight identified by our polyclonal and mAbs.

During the processing of the E7 protein in the Cos-7 transfected cells, the protein is localized at the perinuclear zone by 24 h after being synthesized. This localization is in agreement with a previous report that showed that E7 from HPV-16 was observed in ER, cytoplasm and the nuclear membrane, where E7 was able to activate the α-glucosidase enzyme in an allosteric way [18]. Subsequently, during the expression of the E7 protein in the Cos-7 transfected cells (48 h), the protein was visualized in the nucleus and relocalized into the nucleolus of the cells. These structures have also been reported to be present in CaSki cells, but only during the G2/M cell cycle phase [53]. The nuclear localization of the E7 protein has been reported in several studies [50, 51, 53, 54]; however, this protein does not contain a consensus sequence to be translocated into the nucleus. The translocation of the E7 protein into this compartment has been reported to be through a non-conventional Ran-GTP pathway and an intermediary carrier protein seems to be involved [54]. Alternatively, *in vitro* characterization of E7 protein has demonstrated that formation of dimers and tetramers of E7 depends on pH changes which allow exposure of hydrophobic epitopes [13]. It is also well established that cell compartments are microenvironments that functions at different pHs [55, 56]. It is possible then, that final localization of the different isoforms of E7 in the cell could be drived by exposure of different epitopes due to oligomerization of E7 induced by changes in pH, during their transit through the different cellular compartments.

The low levels of E7 expression of the high risk HPVs, together with the short half-life of this protein, makes it difficult to carry on with biochemical experiments helpful to completely characterize this protein. Most of the information obtained in an effort to biochemically characterize E7 protein has been generated from recombinant E7 protein produced in bacteria or produced under *in vitro* conditions. However, some of the posttranslational modifications of the proteins are predominantly observed in eukaryotic cells. The lack of these modifications would change the molecular weights as well as the net charges of the protein, making it difficult to detect the intermediates or processed forms of the HPV-16 E7 protein that are reported in this paper as we used eukaryotic cell cultures. In this way, 3 forms of HPV-16 E7 proteins were identified with polyclonal and mAbs that showed different molecular weights and different IEPs (E7a1, E7a and E7b). Due to the fact that the different antibodies recognized different epitopes, the E7 protein was recognized in different cellular compartments at different times during its processing.

The E7 protein has been recognized as a multifunctional protein that interacts with a high variety of target proteins. It will be of great interest to identify the complete molecular processing of the E7 protein (other posttranslational modifications) that will allow a better knowledge about the structure and the biological activity of this oncogenic protein and its role in the transformation process.
